# Adverse birth outcomes among native-born and foreign-born mothers in Taiwan: A population-based birth cohort study

**DOI:** 10.1186/1471-2393-12-110

**Published:** 2012-10-13

**Authors:** Laura Wen-Shuan Shiao, Tung-liang Chiang

**Affiliations:** 1 Department of Community and Global Health, Graduate School of Medicine, The University of Tokyo, 7-3-1 Hongo, Bunkyo-ku, Tokyo, Japan; 2 Institute of Health Policy and Management, College of Public Health, National Taiwan University, Room 620, No.17, Hsu-Chow Rd, Taipei, Taiwan

**Keywords:** Low birth weight, Preterm birth, Foreign-born, Socioeconomic position, Epidemiological paradox, Healthy migrant effect.

## Abstract

**Background:**

The number of children born to foreign-born mothers in Taiwan has significantly increased since the 1990s. These foreign-born mothers are mainly from China and Southeast Asia. Children born to foreign-born mothers, according to media reports, are subject to inferior health. This study sought to determine whether socioeconomic disparities in birth outcomes exist between native and foreign-born mothers in Taiwan.

**Methods:**

Analysis data were obtained from the Taiwan Birth Cohort Study of 20,090 nationally representative 6-month-old babies, born in 2005. The data on the babies were divided into two groups, those of foreign-born mothers and those of Taiwanese mothers. The health outcome variables that were examined included two adverse birth outcomes: low birth weight and preterm birth. Multiple logistic regression was used to examine the association between income and foreign-born status, as well as birth outcomes among both groups.

**Results:**

Children of native Taiwanese mothers had a higher prevalence of low birth weight (6.9%) than did children of China-born (4.7%) and Southeast Asia-born mothers (5.2%). The prevalence of preterm birth was also higher among children of native Taiwanese mothers (8.4%) than among children of Southeast Asia-born (7.2%) and China-born mothers (6.3%). Foreign-born status was associated with lower odds of low birth weight among families with a monthly family income < NT$30,000 (adjusted odds ratio (AOR) = 0.24, 95% confidence interval (CI) = 0.14–0.42, *p* < 0.001), and lower odds of preterm birth among families with a monthly family income < NT$30,000 and NT$30,000–69,999 (AOR = 0.63, CI = 0.40–0.99, *p* < 0.05, and AOR = 0.68, CI = 0.53–0.88, *p* < 0.01, respectively). Having a higher monthly family income (NT$70,000+ and NT$30,000–69,999) was associated with lower odds of low birth weight (AOR = 0.59, CI = 0.46–0.77, *p* < 0.001 and AOR = 0.75, CI = 0.60–0.94, *p* < 0.05, respectively) among Taiwanese mothers, but not among foreign-born mothers.

**Conclusion:**

Foreign-born mothers from China and Southeast Asia did not experience worse birth outcomes than native Taiwanese mothers did, regardless of the disadvantaged socioeconomic position of their families.

## Background

Cross-border marriages in the Asia-Pacific region have increased significantly over recent decades. In Japan, the percentage of cross-border marriages had increased from 0.9% in 1980 to 4.3% in 2010 [1]. In South Korea, one tenth of South Korean marriages were to a foreign spouse in 2011 [2]. With the highest rate of cross-border marriages in the Asia-Pacific region [3], the rate in Taiwan peaked in 2003, with 31.9% of registered marriages involving a foreign spouse. The proportion declined to 16.8% in 2006, following the introduction of a more stringent immigration interview system [4]. A similar but significant percentage has continued since.

The number of children born to immigrant mothers, particularly from China and Southeast Asia, has also increased significantly in Taiwan since the 1990s [5]. Although economic development has brought wealth to urban areas, Asian men living in rural areas or those who are less socioeconomically attractive have experienced difficulty seeking wives. Therefore, they seek non-native women, frequently from less-developed areas in Asia. These men face the shared pressure originating from Asian cultural tradition that emphasizes the significance of lineal heritage. In Taiwan, more than 80% of immigrants in cross-border marriages are women, predominantly from mainland China, Vietnam, and Indonesia [6].

Despite a lack of evidence-based information, children born to foreign-born mothers are portrayed negatively as having inferior health [7,8]. Because the total fertility rate of Taiwan became the world’s lowest (0.9 births per woman) in 2010 [9], children born to foreign-born mothers have contributed significantly to the growing share of total newborns (from 5% in 1998 to 10% in 2008) [10].

Previous studies on pregnancy outcomes among immigrant women have yielded heterogeneous results among immigrant groups, as compared to their native counterparts. In the United States, researchers have observed an epidemiological paradox in birth outcomes among foreign-born women of disadvantaged socioeconomic status [11-13], whereas elevated odds of adverse prenatal outcomes was noted among India-born women [14]. In Belgium and France, North African immigrant women from Algeria, Morocco, and Tunisia experienced more favorable birth outcomes than did native women [15]. Several studies have shown the birth outcomes of immigrant women in Taiwan [16-19].

One previous study reported more favorable birth outcomes, including reduced odds of preterm birth, low birth weight, and early neonatal mortality among foreign-born mothers in Taiwan [16]. One hospital-based study conducted in Kaohsiung, Taiwan suggested that native Chinese and Vietnamese mothers have lower odds of preterm birth and heavier babies following adjustments for maternal demographic characteristics and prenatal service [17]. Other previous studies using either county- or country-level birth report data have also confirmed more favorable birth outcomes among foreign-born mothers in Taiwan [16,18,19]. Some predisposing maternal factors to birth outcomes, such as maternal age and pregnancy complications, have been considered in previous studies; however, other critical factors, such as the timing and number of prenatal visits, were not considered [18,19]. Hospital-based studies and studies using data from the Taiwan Birth Reporting Database have not examined in detail the family socioeconomic factors that may contribute to birth outcome disparities.

This study used a nationally representative sample with comprehensive measures to analyze individual data dimensions of the family socioeconomic environment, maternal risk factors, pregnancy complications, prenatal care, and various health variables. Specifically, this study examined the association between family income and disparities in low birth weight and preterm birth among foreign-born and Taiwanese mothers who reside permanently in Taiwan.

## Methods

### Sample

Data for analysis were derived from the Taiwan Birth Cohort Study (TBCS). The TBCS sample used in this study was a nationally representative cohort of 24,200 live births born between January 2005 and December 2005. The TBCS sample was drawn from the Taiwan Birth Reporting Database, using a two-stage stratification random sampling method, with an average sampling rate of 11.7%. The TBCS survey involved face-to-face interviews with mothers or primary caregivers to acquire information on family socioeconomic circumstances, maternal health conditions during pregnancy, use of prenatal care, and the health conditions and development of the infants. Information including birth weight, gestational age, birth order (first born or subsequent births), family place of residence, and maternal age were obtained from birth reports. Survey questions were prepared in languages most comfortable to the respondents, and informed consent was obtained from all participants. A total of 21,248 mothers (87.8%) completed the first-wave survey when their infants were 6 months old.

Births with either parent deceased (n = 21) or those missing information for birth order (n = 17), parent marital status (n = 606), paternal education (n = 175), maternal education (n = 38), family monthly income (n = 73), smoking during pregnancy (n = 25), first-time prenatal care (n = 43), and number of prenatal visits (n = 213) were excluded from this study. Only infants whose fathers were native Taiwanese and whose mothers were either native Taiwanese or immigrants from China or Southeast Asia were included in the study. In total, 1,158 respondents (5.4%) were excluded from this study. The final sample of 20,090 births (94.6%) consisted of 17,441 Taiwan-born (86.8%) and 2,649 (13.2%) foreign-born mothers.

### Variables

The outcome variables of this study were low birth weight and preterm birth. Low birth weight is defined as birth weights less than 2,500 g. Delivery before a gestational age of 37 completed weeks is considered a preterm birth. The data on gestational age were acquired from the Taiwan Birth Reporting Database, from which the sample of the TBCS was drawn. Gestational age is calculated from the first day of the last menstrual period (LMP) to the date of birth with the assistance of obstetric ultrasonography to measure the fetus size.

The independent variable (location of maternal nativity) was assessed by asking mothers their country of origin. One crucial limitation identified in the literature on birth outcomes of immigrant women involves using broad ethnic or racial categories, which fail to capture the heterogeneity of immigrant groups [20]. In Taiwan, mothers who emigrated from China share the same language and similar cultures as Taiwanese mothers do, whereas other Southeast Asian women do not. These social and cultural factors play a vital role in cultural assimilation, which may influence future birth outcomes. Therefore, the mothers examined in this study were categorized for univariate analysis into the following three groups, according to their self-reported country of origin: Taiwan, China, and Southeast Asia. China-born mothers and Southeast Asia-born mothers were later combined into one group for multiple logistic regression.

Other independent variables included infant sex (boy or girl), birth order (first born or subsequent births), plurality (singleton or multiple birth), place of residence (urban or rural), smoking during pregnancy (yes or no), pregnancy complications (yes or no for diabetes, hypertension, pre-eclampsia, placenta previa, and placental abruption), first time use of prenatal care (< 3, 3, and 4+ months of pregnancy), and number of prenatal visits (< 10, 10, 11+ times). Maternal education was measured as years of received formal education categorized into three groups (0–9, 10–12, and 13+ years). Family socioeconomic status was characterized according to family income (average monthly income for the last fiscal year) recoded into three groups (< NT$30,000, NT$30,000–69,999, and NT$70,000+; US$1 equaled NT$32.167 in 2005 [21]). Because maternal education and family income were highly correlated (*r* = 0.60), we used family income to represent family socioeconomic position.

### Statistical Analysis

The distribution of risk factors associated with low birth weight and preterm birth among Taiwan-born, China-born, and Southeast Asia-born mothers was examined. Prevalence of low birth weight, preterm births, and means of birth weight and gestational age were also calculated. Chi-square and *t* tests were then employed to examine the bivariate relationships among maternal nativity, risk factors, and birth outcomes. Pairwise comparisons were employed to compare family and maternal characteristics and birth outcomes between nativity groups. Bonferroni correction was used to counteract multiple comparisons [22]. Because there was no statistical difference in odds for low birth weight and preterm births between China-born and Southeast Asia-born mothers in multiple logistic regression, China-born and Southeast Asia-born mothers were combined into one group. Interaction between maternal nativity and family income was assessed using stratified analysis. The association between maternal nativity and birth outcomes in family income strata, and the association between family income and birth outcomes by maternal nativity was assessed. Multiple logistic regression was further used to examine the association among maternal nativity, family income, and two birth outcomes in unadjusted models, as well as models adjusted for infant sex, birth order, maternal age, singleton birth, place of residence, maternal education, smoking during pregnancy, pregnancy complications, first-time prenatal care, and number of prenatal visits. Data analyses were performed using SAS 9.2 (SAS Institute Inc., Cary, NC, USA, 2002–2008).

The Taiwan Birth Cohort Study was approved by the IRB of Bureau of Health Promotion, the Department of Health and the Directorate-General of Budget, Accounting, and Statistics, Executive Yuan, ROC (No. 94-C3–0940005257).

## Results

### Characteristics of children

Table 1 shows the distribution of selected infant and maternal factors known to influence birth outcomes by maternal nativity. For maternal age, more native Taiwanese mothers were 35 years of age and older (13.6%) than China-born (9.1%) and Southeast Asia-born (3.0%) mothers. A maternal age of less than 20 years was most frequent for Southeast Asia-born mothers (7.0%). Southeast Asia-born mothers were also more likely to be first-time mothers (primipara; 57.3%), followed by China-born (52.6%) and native Taiwanese (49.4%) mothers.

**Table 1:** Maternal and family characteristics of infants by maternal nativity: Taiwan Birth Cohort Study, 2005

**Characteristic**		**Taiwan n = 17441**		**China n = 917**		**Southeast Asia n = 1732**	***p***
	**n**	**%**	**n**	**%**	**n**	**%**	
Infant sex							0.99
Boy	9180	52.6	485	52.9	910	52.5	
Girl	8261	47.4	432	47.1	822	47.5	
Maternal age^abc^							<0.0001^d^
<20 y	225	1.3	0	0.0	121	7.0	
20–34 y	14846	85.1	834	91.0	1539	90.0	
35 y	2370	13.6	83	9.1	52	3.0	
First born^bc^	8620	49.4	482	52.6	993	57.3	<0.0001^d^
Singleton^bc^	16954	97.2	895	97.6	1710	98.7	0.0007^d^
Family monthly income^abc^							
<NT$30,000	1351	7.8	225	24.5	611	35.3	<0.0001^d^
NT$30,000–69,999	9653	55.4	621	67.7	1072	61.9	
NT$70,000+	6437	36.9	71	7.7	49	2.8	
Paternal education^abc^							<0.0001^d^
0–9 y	1666	9.6	247	26.9	733	42.3	
10–12 y	6797	39.0	418	45.6	829	47.9	
13+ y	8978	51.5	252	27.5	170	9.8	
Maternal education^abc^							<0.0001^d^
0–9 y	1161	6.7	479	52.2	1214	70.1	
10–12 y	7194	41.3	338	36.9	430	24.8	
13+ y	9086	52.1	100	10.9	88	5.1	
Place of residence^bc^							<0.0001^d^
Urban	10319	59.2	526	57.4	770	44.5	
Rural	7122	40.8	391	42.6	962	55.5	
Length of stay in Taiwan, y		-		3.3		3.5	
Smoking during pregnancy^abc^	586	3.4	5	0.6	0	0.0	<0.0001^d^
Pregnancy complications							
Diabetes^bc^	430	2.5	15	1.6	8	0.5	<0.0001^d^
Hypertension^ac^	380	2.2	8	0.9	11	0.6	<0.0001^d^
Pre-eclampsia^bc^	141	0.8	4	0.4	1	0.1	0.0012^d^
Placenta previa^ac^	337	1.9	7	0.8	5	0.3	<0.0001^d^
Placental abruption^bc^	79	0.5	4	0.4	0	0.0	0.02
First-time prenatal care^ac^							<0.0001^d^
<3 m	12693	72.8	601	65.5	1143	66.0	
3 m	3922	22.5	210	22.9	405	23.4	
4+ m	826	4.7	106	11.6	184	10.6	
Number of prenatal visits^ac^							<0.0001^d^
<10	2586	14.8	203	22.1	415	24.0	
10	4333	24.8	230	25.1	456	26.3	
11+	10522	60.3	484	52.8	861	49.7	

1 US dollar equaled 32.167 New Taiwan dollars in 2005 [21].

^a^Significant difference at 0.05 level between Taiwan-born and China-born mothers.

^b^Significant difference at 0.05 level between China-born and Southeast Asia-born mothers.

^c^Significant difference at 0.05 level between Taiwan-born and Southeast Asia-born mothers.

^d^Significant after Bonferroni correction. By Bonferroni correction test, a *p* value of < 0.0167 was regarded as significant.

Significant nativity differences in family socioeconomic characteristics were observed. As shown in Table 1, infants of native Taiwanese mothers were most likely to be from high-income families, and with parents who have had higher levels of education. Infants of China-born and Southeast Asia-born mothers had families with similar percentages in the medium income bracket (NT$30,000–69,999), whereas infants of Southeast Asia-born mothers were more likely to have families in the lowest income bracket (35.3%). In total, 51.5% of the fathers had 13 years or more education among native Taiwanese mothers, as compared to 27.5% for China-born mothers and 9.8% for Southeast Asia-born mothers. More than half of the native Taiwanese mothers had 13 years or more of education, whereas one fifth of China-born mothers and one tenth of Southeast Asia-born mothers had attained the same education level of 13 years or more. Infants of native Taiwanese mothers and China-born mothers were more likely to reside in urban areas than those of Southeast Asia-born mothers.

Table 1 lists distributions of smoking during pregnancy, pregnancy complications, and use of prenatal care. Native Taiwanese mothers have a higher percentage of smoking during pregnancy (3.4%) than do their immigrant counterparts (0.6% among China-born mothers and 0% among Southeast Asia-born mothers). Native Taiwanese mothers also have the highest prevalence of all pregnancy complications, such as diabetes (2.5%), hypertension (2.2%), pre-eclampsia (0.8%), placenta previa (1.9%), and placental abruption (0.5%) among the three groups. China-born and Southeast Asia-born mothers had higher percentages of entering first-time prenatal care later than 3 months into pregnancy (11.6% and 10.6%, respectively). Native Taiwanese mothers showed a higher number of prenatal care visits (60.3%), followed by China-born mothers (52.8%) and Southeast Asia-born mothers (49.7%). Except for infant sex (*p* = 0.99), the distributions of maternal age, birth order, singleton birth, family monthly income, parental education, maternal education, place of residence, smoking during pregnancy, pregnancy complications, and prenatal care use were all statistically significantly associated with maternal nativity (*p* < 0.05). After Bonferroni correction for multiple comparisons, maternal age, birth order, singleton birth, family monthly income, parental education, maternal education, place of residence, smoking during pregnancy, pregnancy complications such as diabetes, hypertension, pre-eclampsia, placenta previa, and prenatal care use remained statistically significant. A *p* value of < 0.0167 was regarded as significant (3 tests in total, 0.05/3).

### Birth outcomes

Table 2 presents the birth outcomes of the three maternal groups. Infants of China-born mothers had a higher mean birth weight (3218±456.0 g) than those of native Taiwanese mothers (3102±448.6 g) and Southeast Asia-born mothers (3094±412.3 g). Infants of native Taiwanese mothers were more likely to experience low birth weight (6.9%), as compared to those of Southeast Asia-born mothers (5.2%) and China-born mothers (4.7%). Among all maternal groups, the native Taiwanese group exhibited the lowest mean gestational age (38±1.6 weeks) and highest rate of preterm births (8.4%).

**Table 2:** Birth outcomes of infants by maternal nativity: Taiwan Birth Cohort Study, 2005

**Birth outcome**	**Taiwan**	**China**	**Southeast Asia**	***p***
Mean birth weight	3102±448.6	3218±456.0	3094±412.3	
mean ± SD (g)				
Low birth weight	6.9	4.7^a^	5.2^c^	0.001^d^
(<2500 g), %				
Mean gestational age	38±1.6	39±1.6	39±1.6	
mean ± SD (wk)				
Preterm birth (<37 wk), %	8.4	6.3^a^	7.2	0.02

*p*: test for proportion.

^a^Significant difference at 0.05 level between Taiwan-born and China-born mothers.

^b^Significant difference at 0.05 level between China-born and Southeast Asia-born mothers.

^c^Significant difference at 0.05 level between Taiwan-born and Southeast Asia-born mothers.

^d^Significant after Bonferroni correction. By Bonferroni correction test, a *p* value of < 0.0167 was regarded as significant.

China-born and Southeast Asia-born mothers were more likely to have family incomes of less than NT$30,000 (24.5% and 35.3%, respectively), as compared to native Taiwanese mothers (7.8%; Table 1), whereas foreign-born mothers with the same family income experienced low birth weight (4.0%) and preterm birth (6.2%) less frequently than native Taiwanese mothers did (Table 3).

**Table 3:** The association between maternal nativity and birth outcomes by family income: Taiwan Birth Cohort Study, 2005

**Income**	**Nativity**	**Low birth weight**								**Preterm birth**							
		**n**	**%**	**OR**		**95% CI**	**AOR**^**a**^		**95% CI**	**n**	**%**	**OR**		**95% CI**	**AOR**^**b**^		**95% CI**
<NT$30,000	Taiwan-born	131	9.7	1.00			1.00			128	9.5	1.00			1.00		
n = 2187	Foreign-born	33	4.0	0.38	***	0.26–0.57	0.24	***	0.14–0.42	52	6.2	0.63	**	0.45–0.89	0.63	*	0.40–0.99
NT$30,000–69,999	Taiwan-born	685	7.1	1.00			1.00			835	8.7	1.00			1.00		
n = 11346	Foreign-born	94	5.6	0.77	*	0.62–0.96	0.77		0.58–1.03	122	7.2	0.82	*	0.67–1.00	0.68	**	0.53–0.88
NT$70,000+	Taiwan-born	395	6.1	1.00			1.00			505	7.9	1.00			1.00		
n = 6557	Foreign-born	6	5.0	0.81		0.35–1.84	0.67		0.24–1.86	8	6.7	0.84		0.41–1.73	0.63		0.25–1.55

*p<0.05, **p<0.01, ***p<0.001.

AOR^ab^ : Adjusted odds ratio for infant sex, birth order, maternal age, singleton, place of residence, maternal education, smoking during pregnancy, pregnancy complications, first-time prenatal care, and number of prenatal visits.

1 US dollar equaled 32.167 New Taiwan dollars in 2005 [21].

Table 3 also shows the association between maternal nativity and birth outcomes by family income before and after adjustment for maternal and family characteristics (infant sex, birth order, maternal age, singleton birth, place of residence, maternal education, smoking during pregnancy, pregnancy complications, first-time prenatal care, and the number of prenatal visits). Among mothers with monthly incomes of less than NT$30,000, the immigrant group had lower crude odds of low birth weight (odds ratio (OR) = 0.38, CI = 0.26–0.57, *p* < 0.001) and preterm birth (OR = 0.63, CI = 0.45–0.89, *p* < 0.01) relative to the native Taiwanese group. However, after adjustment for maternal and family characteristics, foreign-born mothers had even lower odds for low birth weight (AOR = 0.24, CI = 0.14–0.42, *p* < 0.001) and preterm birth (AOR = 0.63, CI = 0.40–0.99, *p* < 0.05). Among mothers with a middle monthly income (NT$30,000–69,999), only the immigrant group had lower adjusted odds of preterm birth than did the native Taiwanese group after adjustment for maternal and family characteristics (AOR = 0.68, CI = 0.53–0.88, *p* < 0.01). Among mothers with a high monthly income (> NT$70,000), neither adjusted odds of low birth weight nor adjusted odds of preterm birth was associated with the foreign-born status of mothers.

Table 4 shows the association between family income and birth outcomes by maternal nativity before and after adjustment for infant sex, birth order, maternal age, singleton birth, place of residence, maternal education, smoking during pregnancy, pregnancy complications, first-time prenatal care, and the number of prenatal visits. Native Taiwanese mothers with family incomes of greater than NT$30,000 have lower odds for low birth weight than do mothers with family incomes of less than NT$30,000 after adjustment for family and maternal characteristics (AOR = 0.75, CI = 0.60–0.94, *p* < 0.05 and AOR = 0.59, CI = 0.46–0.77, *p* < 0.001, respectively). However, the association between family income and low birth weight was statistically insignificant among foreign-born mothers. The association between family income and preterm birth is statistically insignificant for income groups among both native Taiwanese and foreign-born mothers.

**Table 4:** The association between family income and birth outcomes by maternal nativity: Taiwan Birth Cohort Study, 2005

**Nativity**	**Income**	**Low birth weight**								**Preterm birth**					
		**n**	**%**	**OR**		**95% CI**	**AOR**^**c**^		**95% CI**	**n**	**%**	**OR**	**95% CI**	**AOR**^**d**^	**95% CI**
Taiwan-born	<NT$30,000	131	9.7	1.00			1.00			128	9.5	1.00		1.00	
n = 17441	NT$30,000–69,999	685	7.1	0.71	***	0.59–0.87	0.75	*	0.60–0.94	835	8.7	0.91	0.74–1.10	1.01	0.81–1.27
	NT$70,000+	395	6.1	0.61	***	0.50–0.75	0.59	***	0.46–0.77	505	7.9	0.81	0.66–1.00	0.90	0.70–1.15
Foreign-born	<NT$30,000	33	4.0	1.00			1.00			52	6.2	1.00		1.00	
n = 2649	NT$30,000–69,999	94	5.6	1.43		0.95–2.15	1.41		0.89–2.25	122	7.2	1.17	0.84–1.64	1.16	0.80–1.68
	NT$70,000+	6	5.0	1.28		0.53–3.12	1.24		0.41–3.77	8	6.7	1.08	0.50–2.33	1.04	0.42–2.60

*p<0.05, **p<0.01, ***p<0.001.

AOR^cd^ : Adjusted odds ratio for infant sex, birth order, maternal age, singleton, place of residence, maternal education, smoking during pregnancy, pregnancy complications, first-time prenatal care, and number of prenatal visits.

1 US dollar equaled 32.167 New Taiwan dollars in 2005 [21].

## Discussion

We examined two birth outcomes: low birth weight and preterm births between immigrant and native Taiwanese mothers by socioeconomic status using nationally representative data of a 2005 birth cohort. By assessing the interaction between foreign-born status and family income, we were able to examine the role of foreign-born status at different family income levels and whether an income gradient exists in low birth weight and preterm births among native Taiwanese and foreign-born mothers. Our results show that foreign-born status plays a protective role against low birth weight and preterm birth among mothers with lower family incomes, whereas this protective role was not observed among mothers with high family income (Table 3). The influence of family income on low birth weight varies among foreign-born mothers and native Taiwanese mothers (Table 4). The gradient between income and low birth weight exists among native Taiwanese mothers; that is, the probability of experiencing low birth weight is higher for mothers with low incomes, whereas no association exists between income and low birth weight among foreign-born mothers. In addition, no association exists between family income and preterm birth among either native Taiwanese or foreign-born mothers. Our findings support the literature on “epidemiological paradox” showing that the association between socioeconomic status and birth outcomes was weaker among foreign-born mothers [13,23].

All foreign spouses are eligible to apply for the National Health Insurance (NHI) 4 months after entering Taiwan. Taiwan's NHI covers at least 10 free prenatal visits (more if necessary), regardless of nationality. As part of Taiwan’s maternal and child care systems, a schedule of prenatal visits and examinations is recommended in multi-language maternal-child handbooks/passports provided to all pregnant women during their first prenatal visit. Although the available prenatal care resources are the same as those offered to native Taiwanese mothers, Chinese and Southeast Asian mothers were less likely to initiate prenatal care during the first 3 months of pregnancy, and also had fewer prenatal visits. In the context of the negative relationship between the use of prenatal care and birth outcomes, adverse birth outcomes were not observed among native Chinese or Southeast Asian mothers of different family income levels (Table 3). The question of why this is so and the significance of prenatal care remains unknown.

A second possible explanation is that foreign-born mothers had healthier lifestyles during pregnancy. Cigarette smoking is known to have adverse effects on pregnancy outcomes [24], and our study showed that foreign-born mothers were less likely to smoke during pregnancy; therefore, they were less likely to have pregnancy complications (Table 1). This suggests that differences in culture and behavior during pregnancy may exist between the two maternal groups.

A third possible explanation may involve the psychosocial characteristics of women born in other countries. Evidence has indicated that social support may be associated with favorable birth outcomes [25]. As the numbers of cross-border marriages and foreign laborers increase, it may not be too difficult for a foreign-born mother in Taiwan to form friendships with other immigrants from the same country to gain the same social support that native Taiwanese mothers receive. Conversely, perceived discrimination and other social barriers may cause stress, which has been found to be a risk factor for adverse birth outcomes [26]. Health status may deteriorate over time as a response to cumulative discrimination. This effect of growing social inequality on women’s health may later affect fetal health [27]. Foreign-born mothers may experience discrimination and stress prior to and during pregnancy, but exposure may be insufficient to result in adverse birth outcomes because of their short stay in Taiwan (the mean length of stay was 3.3 years and 3.5 years for China-born mothers and South Asia-born mothers, respectively). However, additional studies are required to clarify this assumption.

Finally, as consistent with previous studies in the United States and Europe, our results indicate the existence of a healthy migrant effect, indicating that healthier people are more likely to migrate [23,28]. Foreign-born status was significantly associated with lower odds of low birth weight and preterm health in income groups of less than NT$70,000 (Table 3). The results for favorable birth outcomes suggest that foreign-born mothers may have undergone selection processes before they entered Taiwan. According to the immigration regulations of the Ministry of Foreign Affairs in Taiwan, marriage immigrants from China and Southeast Asia must pass a physical examination prior to entering Taiwan. Therefore, the health examination may act as a favorable health selection for foreign-born mothers in Taiwan. This selection may also cancel out the income gradient of low birth weight among foreign-born mothers, leading to the observation of no association between family income and low birth weight among foreign-born mothers.

The strength of this study is described as follows: (1) This study was based on data from the Taiwan Birth Cohort Study, the first and the largest longitudinal birth cohort in Taiwan. (2) This study addressed comprehensive explanatory factors of birth outcomes that may not have been available to previous studies, including birth order, maternal age, family income, maternal education, place of residence, smoking during pregnancy, pregnancy complications, and prenatal care use among infants of foreign-born mothers. (3) Future studies could benefit from following up the birth cohort to provide a clearer understanding of the epidemiological paradox of the salutary birth outcomes observed among infants of foreign-born mothers in Taiwan.

A chi-square goodness-of-fit test used to determine TBCS sample representativeness showed that the TBCS sample was consistent with the population. The distribution of infant sex (*x*^2^ = 0.74, *p* = 0.3896), multiple births (*x*^2^ = 0.37, *p* = 0.8311), birth weight (*x*^2^ = 1.79, *p* = 0.4086), and gestational age (*x*^2^ = 1.20, *p* = 0.5488) were not significantly different between the TBSC sample and the population, except for birthing setting (*x*^2^ = 14.29, *p* = 0.0007). This may be due to the large sample size and additional hospital births in the TBCS sample (68.43% in sample, and 67.30% in population). Therefore, we conclude that the results in the present study may be generalized to the population.

This study was limited by the data being collected by using a self-report procedure. The self-report results may be subject to mistakes, exaggeration, or underreporting resulting from recall or social desirability bias. Using LMP to determine gestational age may also lead to miscalculation resulting from recall bias; however, adjusting gestational age by using obstetric ultrasonography should minimize the bias. The rates of maternal complications in the TBCS are higher than the rates of the same complications in the Taiwan Birth Reporting Database. This difference may be due to recall bias in the TBCS, or to underreporting in the Taiwan Birth Reporting Database. Higher rates of maternal complications in clinical reports [29,30] suggest the underreporting in the Taiwan Birth Database may be the reason. Data on syphilis, a predisposing maternal factor for adverse birth outcomes, were not collected in the TBCS. In previous studies [19,31], foreign-born mothers in Taiwan (including China-born and Southeast Asia-born) were more likely to have had syphilis than native Taiwanese mothers. Although syphilis had an effect on birth weight [31], it was statistically insignificant on preterm birth in Taiwan [19].

## Conclusion

We observed an “epidemiological paradox” and “healthy migrant effect” of birth outcomes in Taiwan: The association between family income and birth outcomes is weaker in foreign-born mothers than in native Taiwanese mothers. However, children born in cross-border marriages are more likely to live in socioeconomically deprived environments. The question of whether a healthier status before birth and continuing throughout a child’s entire life remains unanswered. Further longitudinal studies are required to examine the health disparities among children of cross-border marriages.

## Abbreviations

AOR: Adjusted odds ratio; CI: 95% Confidence interval; TBCS: Taiwan Birth Cohort Study; LMP: Last menstrual period; OR: Odds ratio.

## Competing interests

The authors declare that they have no competing interests.

## Authors’ contributions

LWS and TLC conceived the design of the study, performed the statistical analysis, and drafted the manuscript. Both authors read and approved the final manuscript.

## Pre-publication history

The pre-publication history for this paper can be accessed here:

http://www.biomedcentral.com/1471-2393/12/110/prepub
